# Identification of ABCG2 as an Exporter of Uremic Toxin Indoxyl Sulfate in Mice and as a Crucial Factor Influencing CKD Progression

**DOI:** 10.1038/s41598-018-29208-w

**Published:** 2018-07-24

**Authors:** T. Takada, T. Yamamoto, H. Matsuo, J. K. Tan, K. Ooyama, M. Sakiyama, H. Miyata, Y. Yamanashi, Y. Toyoda, T. Higashino, A. Nakayama, A. Nakashima, N. Shinomiya, K. Ichida, H. Ooyama, S. Fujimori, H. Suzuki

**Affiliations:** 1 Department of Pharmacy, The University of Tokyo Hospital, Faculty of Medicine, The University of Tokyo, Tokyo, Japan; 2 Department of Integrative Physiology and Bio-Nano Medicine, National Defense Medical College, Tokorozawa, Saitama, Japan; 3 Department of Renal Disease, Tsubasa Clinic, Tokyo, Japan; 4 Division of Kidney and Hypertension, Department of Internal Medicine, Jikei University School of Medicine, Tokyo, Japan; 5 Department of Pathophysiology, Tokyo University of Pharmacy and Life Sciences, Tokyo, Japan; 6 Department of Internal Medicine, Ryogoku East Gate Clinic, Tokyo, Japan; 7 Teikyo University Shinjuku Clinic, Tokyo, Japan

## Abstract

Chronic kidney disease (CKD) patients accumulate uremic toxins in the body, potentially require dialysis, and can eventually develop cardiovascular disease. CKD incidence has increased worldwide, and preventing CKD progression is one of the most important goals in clinical treatment. In this study, we conducted a series of *in vitro* and *in vivo* experiments and employed a metabolomics approach to investigate CKD. Our results demonstrated that ATP-binding cassette transporter subfamily G member 2 (ABCG2) is a major transporter of the uremic toxin indoxyl sulfate. ABCG2 regulates the pathophysiological excretion of indoxyl sulfate and strongly affects CKD survival rates. Our study is the first to report ABCG2 as a physiological exporter of indoxyl sulfate and identify ABCG2 as a crucial factor influencing CKD progression, consistent with the observed association between ABCG2 function and age of dialysis onset in humans. The above findings provided valuable knowledge on the complex regulatory mechanisms that regulate the transport of uremic toxins in our body and serve as a basis for preventive and individualized treatment of CKD.

## Introduction

Chronic kidney disease (CKD) is a disease characterized by chronically impaired kidney function and is attributed to various causes. The number of CKD patients has recently increased worldwide^1^, and several clinical studies revealed that CKD progression increases the risk of cardiovascular disease (CVD)^2^. Patients at the advanced stages of CKD, end stage renal disease, require dialysis treatment, which incurs high medical costs. Thus, preventing CKD progression and decreasing the risk of CVD and dialysis should be one of the most important goals in clinical situations.

CKD patients are known to accumulate various uremic toxins in the body as a result of dysfunctional urinary excretion pathways. Recently, the European Uremic Toxins Work Group listed approximately 90 endogenous compounds as uremic toxins^3^. Of these, protein-bound uremic toxins are thought to be especially toxic because they bind strongly to plasma proteins, such as albumin. In turn, dialytic removal is disrupted and toxicities arise because of unremoved toxins in the body^4^. Therefore, maintenance of the excretion ability for protein-bound uremic toxins is important for CKD therapy, and there is an urgent need to elucidate the molecular mechanisms that mediate (urinary) excretion of uremic toxins.

Since protein-bound uremic toxins strongly bind to plasma proteins, glomerular filtration cannot efficiently remove toxins and transporter-mediated tubular secretion plays an important role in the excretion process. Tubular secretion is comprised of two essential transmembrane pathways that facilitate transport from blood to renal tubular cells and renal tubular cells to the tubular lumen (urine). To date, two putative transporters that mediate blood-to-cell transport have been identified, namely, the organic anion transporter (OAT) 1/SLC22A6 and OAT3/SLC22A8^5^. However, the apical transporter(s) that mediates cell-to-urine transport of uremic toxins remains to be identified.

Recently, we and another group reported that the ATP-binding cassette transporter subfamily G member 2 (ABCG2)/breast cancer resistance protein (BCRP) mediates physiological secretion of urate and that hereditary dysfunction of ABCG2 increases the risk of hyperuricemia and gout^6,7^. In addition, our experiments using *Abcg2*-knockout mice demonstrated that Abcg2-mediated urate secretion is essential for physiological maintenance of urate homeostasis^8^. Moreover, ABCG2 has been reported to function as an apical efflux transporter not only in the kidney, but also in the small intestine and liver^9^. In addition, ABCG2-mediated transport of estrone sulfate was reported to be inhibited by several kinds of uremic toxins^10^. From these pieces of information, we hypothesized that ABCG2 plays an important role in the physiological transport of uremic toxins under CKD conditions and subsequently performed *in vivo* studies using *Abcg2*-knockout mice.

## Results

### *Abcg2*-knockout mice with adenine-induced CKD have lower survival rates

**Figure 1:**
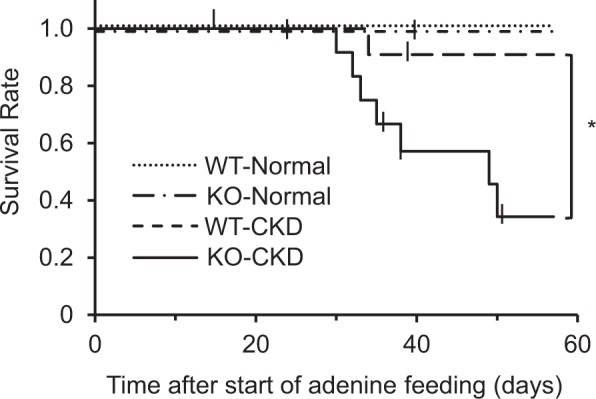
Survival rates of wild-type and *Abcg2*-knockout mice with experimental chronic kidney dysfunction. Kaplan-Meier curves of the survival rates of wild-type (WT) and *Abcg2*-knockout (KO) mice fed with 0.2% adenine-containing or control FR-1 diet (*n* = 6–12) are shown. Dotted and chained lines indicate WT and KO mice fed with control diet (WT-Normal and KO-Normal, respectively), while dashed and solid lines indicate WT and KO mice with chronic kidney disease (CKD) condition, fed with 0.2% adenine-containing diet (WT-CKD and KO-CKD, respectively). *P < 0.05 (WT-CKD vs. KO-CKD, log-rank test).

To construct the CKD mouse model, wild-type (WT) and *Abcg2*-knockout (KO) mice were fed with 0.2% adenine (w/w) as previously reported^11^. Surprisingly, only 31% of *Abcg2*-knockout mice survived after 57 days of feeding with 0.2% adenine (KO-CKD mice), whereas 91% of the wild-type mice with CKD (WT-CKD mice) and 100% of mice with both genotypes subjected to normal feeding survived (Fig. 1). These results suggested that ABCG2 function influences the exposure of mice to uremic toxins. Thus, we measured the plasma concentrations of uremic toxins in wild-type and *Abcg2*-knockout mice.

### Uremic toxins were accumulated in *Abcg2*-knockout mice with CKD

**Figure 2:**
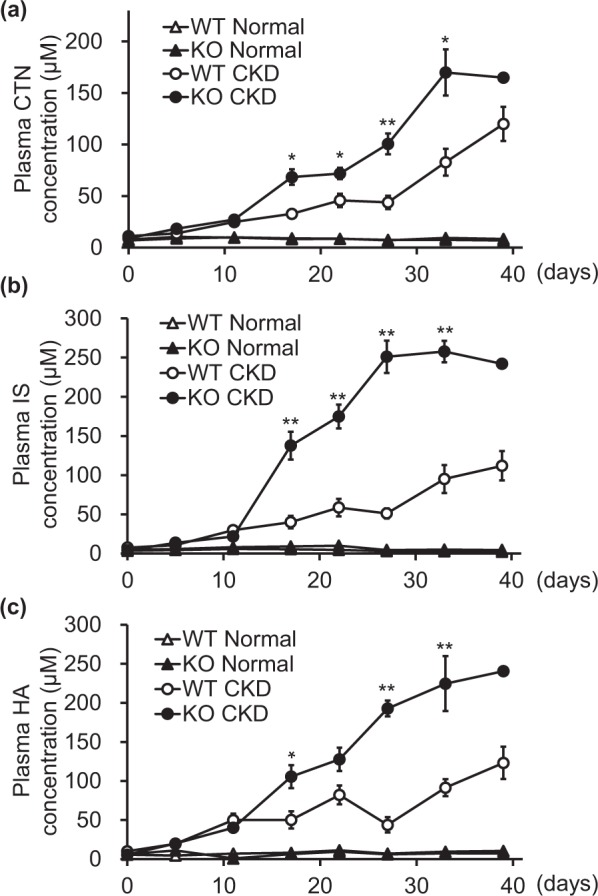
Time profiles of uremic toxins in wild-type and *Abcg2*-knockout mice with or without experimental chronic kidney dysfunction. Time profiles of creatinine (CTN, panel a), indoxyl sulfate (IS, panel b), and hippuric acid (HA, panel c) in wild-type (WT)-Normal (open triangles), *Abcg2*-knockout (KO)-Normal (closed triangles), WT-CKD (open circles), and KO-CKD (closed circles) mice. Data are presented as mean ± S.E. *P < 0.05 and **P < 0.01 (WT-CKD vs. KO-CKD, unpaired Student’s *t*-test).

First, we measured the plasma concentrations of creatinine, a well-established biomarker for kidney function. As shown in Fig. 2a, creatinine concentrations gradually increased in both WT-CKD and KO-CKD mice but were higher in KO-CKD mice compared to those in WT-CKD mice (2.3-fold on day 27). On the other hand, both wild-type and *Abcg2*-knockout mice subjected to normal feeding showed no increase in creatinine levels (Fig. 2a). These results indicated that loss of ABCG2 function causes more severe kidney dysfunction under CKD-induced conditions as a result of accumulation of uremic toxin(s), which should be substrates of ABCG2-mediated transport. Therefore, we performed metabolomics analysis to compare the plasma specimens obtained from the wild-type and *Abcg2*-knockout mice fed with adenine-containing diet for 26 days.

Exhaustive analysis of anionic chemicals (m/z: 100–500) with liquid chromatograph-mass spectroscopy (LC-MS) found 33 putative peaks whose intensities were more than 2.5-fold higher in *Abcg2*-knockout mice. We compared the observed exact masses of the putative peaks with the theoretical monoisotopic masses of 15 small molecule uremic toxins, whose plasma concentrations were reported to be increased more than 10-fold in uremic patients compared with healthy controls^12^. Results showed that two putative peaks corresponded to two known compounds (indoxyl sulfate and hippuric acid) (Supplementary Table S1).

Therefore, plasma concentrations of indoxyl sulfate and hippuric acid were measured using liquid chromatograph-tandem mass spectroscopy (LC-MS/MS). Although the CKD condition increased plasma concentrations of the two compounds in both wild-type and *Abcg2*-knockout mice, *Abcg2*-knockout mice showed stronger responses compared with wild-type mice for both indoxyl sulfate and hippuric acid (4.9-fold and 4.4-fold for indoxyl sulfate and hippuric acid, respectively, on day 27) (Fig. 2b,c). These results suggested that ABCG2 have an ability to transport indoxyl sulfate and/or hippuric acid.

### ABCG2 mediates ATP-dependent transport of indoxyl sulfate

**Figure 3:**
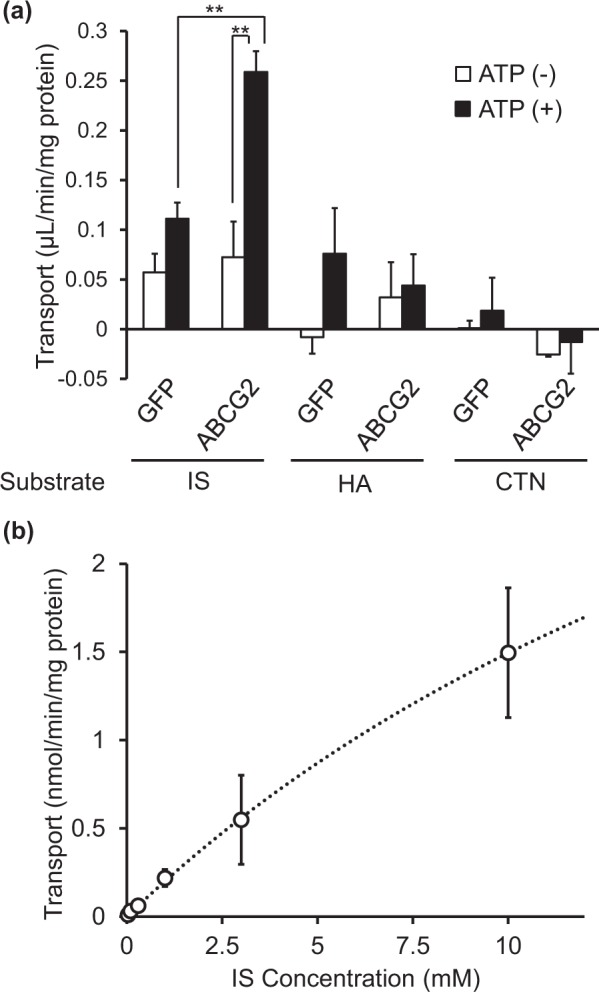
ABCG2-mediated transport of uremic toxins. (**a**) *In vitro* ATP-dependent transport activities of indoxyl sulfate (IS), hippuric acid (HA), and creatinine (CTN) by membrane vesicles prepared from HEK293A cells expressing Myc-human ABCG2 or EGFP. The substrate concentration of IS and HA was 30 µM and that of CTN was 300 µM. Open and closed columns indicate values in the absence and presence of ATP, respectively (mean ± S.E., *n* = 3–4 for each experimental condition). **P < 0.01 (non-repeated ANOVA followed by Student-Newman-Keuls test). (**b**) Concentration-dependent transport of IS by ABCG2 vesicles. Symbols and bars indicate mean and S.E., respectively (*n* = 3–4), and the dotted line indicates fitting line. Each value was calculated by subtracting the transport activity in the absence of ATP from that in the presence of ATP.

To determine whether these uremic toxins are ABCG2 substrates, we performed *in vitro* transport assays using plasma membrane vesicles prepared from ABCG2-expressing cells. ATP-dependent transport of indoxyl sulfate was observed in ABCG2-expressing vesicles, whereas no significant differences were observed for hippuric acid and creatinine in the presence or absence of ATP (Fig. 3a). Moreover, a concentration-dependent transport experiment for indoxyl sulfate revealed that ABCG2 mediates ATP-dependent transport of indoxyl sulfate, with a K_m_ of 25.3 mM and V_max_ of 5.27 nmol/min/mg protein (Fig. 3b).

### *Abcg2*-knockout mice with CKD showed slower elimination of indoxyl sulfate

**Figure 4:**
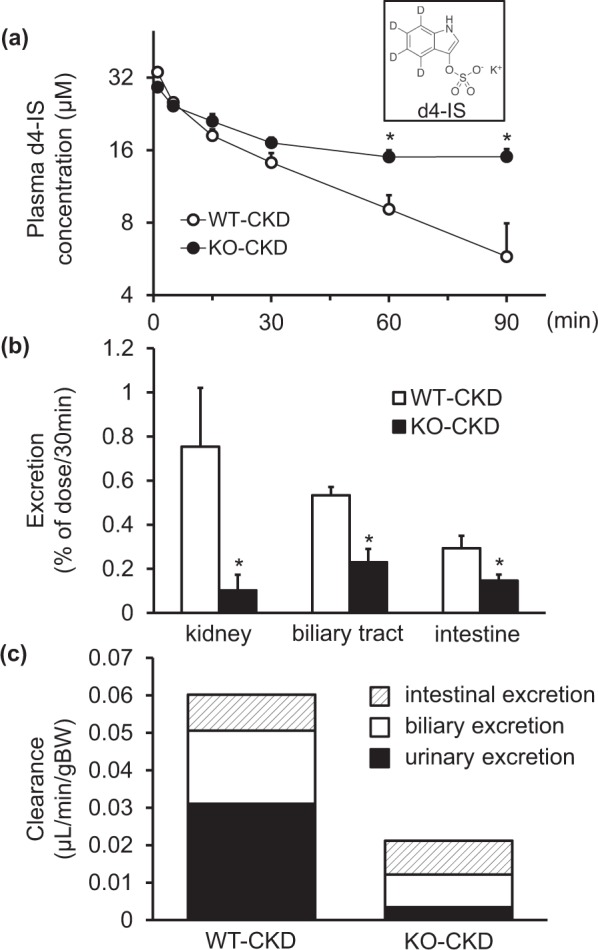
Plasma profiles and excretion of indoxyl sulfate in wild-type and *Abcg2*-knockout mice with chronic kidney dysfunction. (**a**) Plasma concentration-time profiles of deuterium-labeled indoxyl sulfate (d4-IS) in wild-type (WT)-CKD (open circles) and *Abcg2*-knockout (KO)-CKD (closed circles) mice. Symbols and bars indicate mean and S.E., respectively (*n* = 4). KO-CKD mice showed significantly longer t_1/2_ (206.4 ± 104.1 min vs. 48.3 ± 25.0 min, *P* < 0.05) and higher AUC_inf_ (5.89 ± 2.95 mM·min vs. 1.67 ± 0.74 mM·min, *P* < 0.05) compared with those of WT-CKD mice. (**b**) Cumulative excreted amounts of d4-IS (% of dose) via the kidney, biliary tract, and intestine until 30 min after intravenous administration to WT-CKD and KO-CKD mice. Open and closed columns indicate data corresponding to WT-CKD and KO-CKD mice, respectively. Data are presented as mean ± S.E. (*n* = 5–9). *P < 0.05 (unpaired Student’s t-test). (**c**) Apparent clearance of d4-IS in each excretion pathway. Closed, open, and shaded columns indicate urinary, biliary, and intestinal excretion clearances, respectively. Clearances were calculated by dividing the cumulative excreted amount of d4-IS (0–30 min) by the plasma AUC of d4-IS (0–30 min) and normalized by the body weight (BW).

To elucidate the mechanisms responsible for the accumulation of indoxyl sulfate in KO-CKD mice (Fig. 2b), deuterium (d4)-labeled-indoxyl sulfate (d4-IS) was intravenously administered to WT-CKD and KO-CKD mice, and the time profile for plasma concentration of d4-IS was determined using LC-MS/MS (Fig. 4a). Although no significant changes in d4-IS concentration were observed at 1, 5, 15, and 30 min after administration, d4-IS concentrations were significantly higher at 60 and 90 min after administration in KO-CKD mice. Correspondingly, the calculated elimination half-life (t_1/2_) was significantly longer in KO-CKD mice compared with that in WT-CKD mice (206.4 min vs 48.3 min, respectively), and the calculated area under the time-concentration curve (AUC) from 0 min to time infinity (AUC_inf_) was markedly higher in KO-CKD mice (5.89 mM·min vs 1.67 mM·min, respectively). Based on the above results, we performed further *in vivo* experiments to investigate the mechanisms responsible for the slower elimination of d4-IS in *Abcg2*-knockout mice.

### Indoxyl sulfate excretion was decreased in *Abcg2*-knockout mice with CKD

The accumulated amounts of d4-IS excreted into the urine, bile, and intestinal fluid were measured in WT-CKD and KO-CKD mice. Less than 1% of the administered d4-IS was excreted into urine up to 30 min in WT-CKD mice (Fig. 4b), which was remarkably lower than the amount excreted into urine of WT-Normal mice in the same time period (38.5 ± 10.3%), which suggested impaired renal secretion of indoxyl sulfate in the CKD condition. Importantly, urinary excretion of d4-IS in KO-CKD mice was considerably lower than that in WT-CKD mice (0.75% vs. 0.10%, respectively) (Fig. 4b). Moreover, biliary and intestinal excretion of d4-IS was also lower in KO-CKD mice compared to that in WT-CKD mice (Fig. 4b). Sum of clearance for each pathway was calculated to be decreased to approximately one thirds in KO-CKD mice (Fig. 4c), which is consistent with the slower elimination of plasma d4-IS in KO-CKD mice (Fig. 4a).

## Discussion

Our findings revealed that *Abcg2*-knockout mice were susceptible to CKD and had lower survival rates (Fig. 1), impaired kidney function (Fig. 2a), and higher uremic toxin levels in plasma under the CKD condition (Fig. 2b,c). These results are consistent with our previous studies on human patients, which showed that impaired ABCG2 function due to genetic combination of dysfunctional variants is linked to earlier age of dialysis onset (Supplementary Table S2)^13,14^. Taken together, our results indicated that ABCG2 function plays a crucial role in CKD progression, in addition to its previously reported roles in hyperuricemia/gout^6,8^, Parkinson’s disease^15^, and cancer^16^.

Transport experiments using plasma membrane vesicles showed that ABCG2 mediates ATP-dependent transport of indoxyl sulfate, but not hippuric acid or creatinine (Fig. 3a). In addition, indoxyl sulfate levels showed more drastic increases in KO-CKD mice than that of creatinine, which cannot be simply explained by impaired kidney function estimated by plasma creatinine levels (Fig. 2a,b). Together with the toxicities of indoxyl sulfate, which has been reported as an oxidative stress inducer and kidney fibrosis stimulator^17^, our findings suggested that loss of Abcg2-mediated indoxyl sulfate efflux is associated with higher susceptibility of *Abcg2*-knockout mice to kidney dysfunction.

To our knowledge, there is no report comparing the differences of indoxyl sulfate production between normal and kidney dysfunctional conditions or between the wild-type and *Abcg2*-knockout mice. However, since pharmacokinetic profiles of the infused d4-IS were comparable to that of endogenous indoxyl sulfate (Figs 2 and 4), higher plasma d4-IS concentrations in KO-CKD mice should be explained by the lower excretion ability of indoxyl sulfate in *Abcg2*-knockout mice.

The calculated K_m_ value for ABCG2-mediated transport of indoxyl sulfate was 25.3 mM (Fig. 3b). Since reported plasma concentrations of indoxyl sulfate (Table S1), including results shown in Fig. 2b, did not increase to the millimolar order in mice and human CKD conditions, the K_m_ value indicated that ABCG2-mediated indoxyl sulfate transport is not saturated under (patho)physiological conditions and that ABCG2 can maintain its transport activity even under high concentrations of indoxyl sulfate. This type of high-capacity transport property is similar to that of ABCG2-mediated urate transport (K_m_ of 8.24 mM)^6^, which is also unsaturated under (patho)physiological conditions. On the other hand, other reported ABCG2 substrates, such as estrone sulfate, 4-methylumbelliferone sulfate, and 6-hydroxy-5,7-dimethyl-2-methylamino-4-(3-pyridylmethyl) benzothiazole (E3040) sulfate, have low capacity with K_m_ values of ~20 μM^18^. Further detailed studies are required to clarify the mechanisms responsible for the higher K_m_ value of indoxyl sulfate compared with other sulfate conjugates.

OAT1 and OAT3 have been reported to function as indoxyl sulfate transporters^5^. Both OAT1 and OAT3 mediate cellular transport of various organic anions in the basolateral membrane of proximal tubular cells in the kidney^19^ and can also transport indoxyl sulfate^5^. In addition, *Oat1*-knockout mice showed dramatically higher plasma concentrations of indoxyl sulfate, highlighting the major role of OAT1 in the uptake process from the blood to the kidney^20^. Organic anion transporting polypeptide 1B3 (OATP1B3/SLCO1B3/SLC21A8) was also reported to transport indoxyl sulfate^21^, suggesting its function as an indoxyl sulfate transporter in the basolateral membrane of hepatocytes. On the other hand, the apical transporter(s) for indoxyl sulfate have not been reported. Therefore, the present study is the first to demonstrate that ABCG2 is the primary apical transporter that mediates the excretion of indoxyl sulfate from the body. Comparison of the sum of clearance values between WT-CKD and KO-CKD mice suggested that ABCG2 acts as a major efflux transporter of indoxyl sulfate, although limited indoxyl sulfate excretion was still observed in KO-CKD mice (Fig. 4c). ABCC4/multidrug resistance-associated protein 4 (MRP4) is a candidate transporter responsible for this pathway because ABCC4 is known to function as an efflux pump on the brush border membrane of epithelial cells in the kidney and small intestine^22^. Furthermore, ABCC4 exhibits similar substrate specificity with ABCG2^23^ and was shown to be inhibited by indoxyl sulfate in a concentration-dependent manner^10^.

Most uremic toxins are excreted into the urine, although excretion via this pathway is known to be dramatically reduced under CKD conditions. In fact, d4-IS administration into CKD-induced mice showed that the excreted amounts of d4-IS into bile and intestinal lumen were relatively close to the amounts excreted into the urine (Fig. 4b,c). Under these conditions, indoxyl sulfate excretion into all three pathways were significantly lower in *Abcg2*-knockout mice (Fig. 4b,c), indicating the physiological significance of ABCG2-mediated indoxyl sulfate transport not only in the kidney but also in the liver and small intestine. These findings are consistent with previous reports on *Abcg2*-knockout mice that demonstrated Abcg2-mediated excretion of edaravone sulfate^24^ and E3040 sulfate^25^ into the urine, of urate^8^ and 4-methylumbelliferone sulfate^26^ from the small intestine, and of topotecan^27^ and fluoroquinolones^28^ into the bile. In clinical situations, impaired ABCG2 function may cause lower tolerance against kidney disease caused by reduced ABCG2-mediated excretion of uremic toxins into the kidney, liver, and small intestine, which can in turn accelerate the progression of kidney dysfunction and ultimately lead to dialysis (see Supplementary Table S2)^13,14^.

In this study, we showed that ABCG2 mediates the physiological transport of indoxyl sulfate, a major uremic toxin, and plays a crucial role in its excretion. In addition, our findings revealed that genetic dysfunction of ABCG2 in mice results in lower survival rates against the CKD-inducible condition. These results are consistent with our previous findings in humans, which showed the significant correlation between ABCG2 dysfunction and earlier age of hemodialysis onset. Our findings provided the initial step in elucidating the complex regulatory mechanisms responsible for the excretion of uremic toxins in our body, and this new knowledge can be utilized for the prevention and individualized treatment of CKD.

## Methods

### Chemicals

Indoxyl sulfate potassium salt, ATP, adenosine monophosphate (AMP), creatine phosphate disodium salt tetrahydrate, and creatine phosphokinase type I from rabbit muscles were obtained from Sigma Aldrich (St. Louis, MO). d4-IS potassium salt was purchased from Toronto Research Chemicals (North York, Canada). Adenine, hippuric acid, and creatinine were obtained from Wako Pure Chemical Industries (Osaka, Japan). All other chemicals used were commercially available and were of reagent or analytical grade.

### Experimental animals

WT mice and *Abcg2*-knockout mice (FVB.129P2-Abcg2, Taconic, Hudson, NY) with FVB background, which were described in our previous report^8^, were used in this study. Mice were housed in temperature- and humidity-controlled animal cages with a 12-h dark/light cycle and were free access to water and animal chow (FR-1, Funabashi-Farm, Chiba, Japan). All animal experiments were carried out after approval by the Animal Studies Committee of The University of Tokyo and conducted in accordance with the statement from the local government authority.

### Survival analysis of adenine-induced chronic kidney disease model mice

Adenine-induced CKD model mice were constructed according to previously described methods^11^. Briefly, approximately seven-week-old male WT and KO mice were fed with powdered FR-1 diet supplemented with either 0.2% (w/w) or 0% adenine. Then, the survival of WT and KO mice fed with 0.2% (w/w) adenine-containing diet (WT-CKD mice (n = 12) and KO-CKD mice (n = 12), respectively) or adenine-free diet (WT-Normal mice (n = 6) and KO-Normal mice (n = 6), respectively) were followed-up until 57 days after start of the study diet. Survival rates were analyzed using Kaplan-Meier method, in which deaths from non-renal causes were censored. During the follow-up period, blood samples were drawn from the jugular vein approximately every 5 days to monitor the plasma concentrations of uremic toxins.

### Comprehensive analysis of anionic compounds in mouse plasma

We performed a comprehensive analysis of plasma anionic compounds in KO and WT mice fed with adenine-containing diet for 26 days. A Thermo Scientific Q Exactive Orbitrap mass spectrometer (ThermoFisher Scientific K.K., Yokohama, Japan) coupled with a DIONEX Ultimate 3000 Rapid Separation LC system (ThermoFisher Scientific K.K.) was used for the analysis.

After protein precipitation with four volumes of methanol, 2-µL aliquots of purified plasma samples were separated on a Syncronis aQ column (5 µm, 2.1 × 100 mm, ThermoFisher Scientific K.K.). Separation was performed in linear gradient elution mode with 0.1% formic acid in water (solvent A) and 0.1% formic acid in acetonitrile (solvent B) at a flow rate of 0.3 mL/min. The gradient program is summarized in Supplementary Table S3. The column temperature was set at 40 °C, and ionization was performed using a heated electrospray ionization probe in negative ion mode according to our previous study^29^. All plasma samples were analyzed in duplicate.

Detection was performed using a Q Exactive Orbitrap mass spectrometer controlled using Excalibur software (ThermoFisher Scientific K.K.), and exact masses were calculated using the Qualbrowser program (ThermoFisher Scientific K.K.). Full MS scans were operated in full spectrum acquisition with a scan range of m/z 100–500. Mass spectra were analyzed using SIEVE software version 2.1 (ThermoFisher Scientific K.K.), and the ion peaks satisfying the following criteria were selected: (1) signal intensity is >500,000; (2) within-assay coefficient of variation of signal intensity is lower than 30%; and (3) signal intensity in KO-CKD plasma is higher by more than 2.5-fold compared to that of WT-CKD plasma. Then, the observed exact masses of selected ion peaks were compared with those of the theoretical monoisotopic masses of 15 small molecule (MW <500) uremic toxins whose plasma concentrations were reported to be increased by more than ten-fold in uremic patients relative to healthy controls^12^.

### Vesicle transport assay

The membrane vesicles were prepared from HEK293A cells infected with enhanced green fluorescent protein (EGFP) or Myc human ABCG2 expressing adenovirus as described previously^30^. The vesicle transport assay was performed using rapid filtration technique^6,29,31^ (see Supplementary Information online for detailed protocol).

The amount of substrate (indoxyl sulfate, hippuric acid, and creatinine) taken up into the vesicles was measured using LC-MS/MS. Uptake activity was calculated according to the following equation: uptake activity [μL/min/mg protein] = (amount of substrate taken up into the vesicles [pmol/mg protein])/(substrate concentration in buffer [μM] × incubation time (5 [min])).

ATP-dependent uptake of substrates was calculated by subtracting the uptake activity in the absence of ATP from the uptake activity in the presence of ATP.

To investigate the concentration-dependent transport of indoxyl sulfate, vesicle transport assay was conducted with various indoxyl sulfate concentrations (0.03, 0.1, 0.3, 1, 3, and 10 mM). The uptake velocity of indoxyl sulfate was determined by dividing the amount of indoxyl sulfate taken up into vesicles by the incubation time (5 min), and the ATP-dependent uptake velocity was calculated by subtracting the uptake velocity in the absence of ATP from the uptake activity in the presence of ATP. Results were fitted to Michaelis-Menten kinetics to estimate the K_m_ and V_max_ values.

### Kinetic analysis of d4-IS in KO and WT mice with adenine-induced CKD

To investigate the *in vivo* kinetics of indoxyl sulfate in KO and WT mice with adenine-induced CKD, we used d4-IS to discriminate between the administered and endogenous indoxyl sulfate. KO and WT mice fed with adenine containing diet for 25–30 days were used for the kinetic study.

After anesthetization with urethane, d4-IS (3.3 nmol/g) was administrated intravenously, and the blood samples were drawn from the jugular vein at 1, 5, 15, 30, 60, and 90 min after administration. All blood specimens were centrifuged for 10 min at 20,000 g, 4 °C immediately after sampling, and stored at −80 °C until analysis. Plasma concentrations of d4-IS were measured as described below, and the t_1/2_ and the AUC_inf_ was calculated (see Supplementary Information for detail).

To investigate biliary and urinary d4-IS excretion, the cystic duct was ligated, and the bile duct was cannulated using a Teflon catheter (UT-03, Unique Medical) under urethane anaesthetization. Bile specimens were collected for 30 min, and mice were sacrificed. Urine specimens were collected directly from the bladder using a 25 G needle to evaluate urinary d4-IS excretion. Bile and urine specimens were weighed and subjected to the d4-IS assay. The cumulative amount of unchanged d4-IS excreted into urine and bile was calculated by multiplying the d4-IS concentration by the sample volume. Data were presented as percent of dose.

Intestinal excretion of d4-IS was investigated following a previously described intestinal perfusion method^8^. Briefly, mice were anaesthetized with urethane and subsequently cannulated with polyethylene tubing (SurFlow-Flash 16 G × 2 inches, Terumo, Tokyo, Japan) at the upper duodenum and the middle jejunum to create an intestinal loop at the upper half of the intestine. After removing the intestinal contents by slow infusion with saline and air, saline was introduced into the intestinal loop, and both ends of the loop were closed with syringes for 30 min after intravenous d4-IS administration. Then, saline in the loop was collected using syringes, and d4-IS concentrations were measured. Cumulative intestinal d4-IS excretion was calculated based on the following equation and presented as percent of dose: Cumulative intestinal d4-IS excretion [nmol] = d4-IS concentration in the intestinal loop [μM] × volume of efflux buffer in the intestinal loop [mL] × (length of whole small intestine [cm])/(length of intestinal loop [cm]).

The apparent urinary, biliary, and intestinal d4-IS clearances were calculated by dividing the cumulative amount of d4-IS excreted into each pathway within 0–30 min by the plasma AUC of d4-IS for the same time period, which was calculated using the trapezoidal rule.

### Determination of indoxyl sulfate, d4-IS, hippuric acid, and creatinine levels in biological specimens

An LC-MS/MS system consisting of an ACQUITY UPLC^®^ instrument coupled with a Xevo TQ-S triple-quadrupole MS/MS system (Waters Corp., Milford, MA) was used to measure the concentrations of indoxyl sulfate, d4-IS, hippuric acid, and creatinine in the biological specimens.

After addition of the internal standard (labetalol for creatinine and penicillin G for indoxyl sulfate, d4-IS, and hippuric acid), specimens were purified by protein precipitation with four volumes of methanol, and the 5–10 µL aliquots of the clear supernatant were subjected to LC-MS/MS analysis. In some cases, specimens were diluted or concentrated before injection. The chromatographic conditions and gradient programs are summarized in Supplementary Table S4, and analytes were monitored in multiple reaction monitoring (MRM) mode. The monitoring parameters (m/z of precursor and product ions, ionization mode and retention time) are shown in Supplementary Table S5. Peak analyses were performed using MassLynx NT software version 4.1 (Waters Corp.).

### Statistical Analysis

Differences in survival rates were compared using log-rank test. Unpaired Student’s *t*-test and Student-Newman-Keuls test following non-repeated ANOVA were used for single and multiple comparisons, respectively. Throughout the study, *P* < 0.05 was considered as statistically significant.

## Electronic supplementary material

Supplementary Information
