# Supplementary material for: Identification of ABCG2 as an Exporter of Uremic Toxin Indoxyl Sulfate in Mice and as a Crucial Factor Influencing CKD Progression
Source: Sci Rep. 2018 Jul 24;8:11147. doi: 10.1038/s41598-018-29208-w (PMC6057959; doi:10.1038/s41598-018-29208-w)
Supplement: Supplementary file 1 — Supplementary Information [file 41598_2018_29208_MOESM1_ESM.pdf]

Supplementary Information

Identification of ABCG2 as an Exporter of Uremic Toxin Indoxyl Sulfate in Mice and as a Crucial Factor Influencing CKD Progression.

T. Takada[1,*,+], T. Yamamoto[1,+], H. Matsuo[2,+], J. K. Tan[1,+], K. Ooyama[3], M. Sakiyama[2], H. Miyata[1], Y. Yamanashi[1], Y. Toyoda[1], T. Higashino[2], A. Nakayama[2], A. Nakashima[4], N. Shinomiya[2], K. Ichida[5], H. Ooyama[6], S. Fujimori[7], H. Suzuki[1,*]

[1] Department of Pharmacy, The University of Tokyo Hospital, Faculty of Medicine, The University of Tokyo, Tokyo, Japan.

[2] Department of Integrative Physiology and Bio-Nano Medicine, National Defense Medical College, Tokorozawa, Saitama, Japan.

[3] Department of Renal Disease, Tsubasa Clinic, Tokyo, Japan.

[4] Division of Kidney and Hypertension, Department of Internal Medicine, Jikei University School of Medicine, Tokyo, Japan.

[5] Department of Pathophysiology, Tokyo University of Pharmacy and Life Sciences, Tokyo, Japan.

[6] Department of Internal Medicine, Ryogoku East Gate Clinic, Tokyo, Japan.

[7] Teikyo University Shinjuku Clinic, Tokyo, Japan.

[*] To whom correspondence should be addressed: E-mail: tappei-tky@umin.ac.jp, suzukihi-tky@umin.ac.jp

[+] These authors contributed equally to this work.

This document contains following items:

- Experimental protocol of vesicle transport assay (Page 1).
- Procedure for calculation of pharmacokinetic parameters of d4-IS (Page 2).
- Supplementary Tables S1-S5 (Pages 3-7).

Vesicle transport assay

The ATP-dependent uptakes of indoxyl sulfate, hippuric acid, and creatinine into the membrane vesicles expressing ABCG2 were evaluated using rapid filtration technique as described below.

In a 1.5 mL polypropylene microtube, 16 μL of the transport buffer (10 mM Tris-HCl, 250 mM sucrose, and 10 mM $MgCl_2$, pH 7.4) containing 5 mM ATP or AMP, ATP regenerating system (10 mM creatine phosphate and 100 mg/L of creatine phosphokinase), and substrates (30 μM indoxyl sulfate and hippuric acid or 300 μM creatinine) were pre-incubated at 37°C for 15 min. Then, 4 μL of the membrane vesicle suspension (6.25 mg protein/mL) was rapidly mixed and incubated for 5 min at 37°C. The reaction was terminated by the addition of 1 mL of stop buffer (10 mM Tris-HCl, 250 mM sucrose, and 100 mM NaCl, pH 7.4) and filtered through the glass microfiber filter (0.7 μm, Whatmann, Little Chalfont, UK) pre-rinsed with 50 mM indole acetic acid to minimize non-specific binding. Then, the filter was washed twice with 5 mL of stop buffer, and the substrate remaining on the filter was extracted using 1 mL of methanol. To determine the non-specific binding of the substrate to the glass filter (filter back), we also performed the uptake assay without the membrane vesicles.

Methanol extracts were evaporated using a centrifugal vacuum concentrator (SpeedVac, Sakuma Industries, Chiba, Japan) and subsequently reconstituted in 60 μL of 10% acetonitrile containing the internal standard (labetalol and penicillin G). Then, 5 μL of samples were subjected to LC-MS/MS analysis with the same condition for plasma specimens.

- **Determination of pharmacokinetic parameters of d4-IS**

A model independent analysis was applied for the determination of pharmacokinetic parameters ($t_{1/2}$ and AUC_{inf}) of d4-IS.

The $t_{1/2}$ was calculated by dividing 0.693 by the elimination rate constant, which was calculated from the last three (*i.e.* 30, 60, and 90 min after administration) natural-log-transformed concentration data points using linear regression.

The AUC_{inf} was calculated using the following equation: $AUC_{inf} = AUC_{0\text{-}90min} + AUC_{90min\text{-}inf}$, where $AUC_{0\text{-}90min}$ and $AUC_{90min\text{-}inf}$ indicate the AUC from time 0 to 90 min and 90 min to infinity time, respectively. The $AUC_{0\text{-}90min}$ was calculated based on the linear trapezoidal rule, while the $AUC_{90min\text{-}inf}$ was calculated by integrating the regression lines described above from 90 min to time infinity.

Table S1. List of small molecule uremic toxins showing greater than ten-fold increase in plasma concentration in uremic patients and putative corresponding peaks detected in this study.

Uremic toxins	Concentration in humans [μM][a]		Ratio[a]	Compositional formula	Theoretical monoisotopic mass[b]	Observed m/z	RT[c]	Ratio[d]
	Normal	Uremic						
Phenylacetic acid	<10.3	3,435	334	$C_8H_8O_2$	135.0446	N.D.		
Neopterin	0.005	0.33	60.3	$C_9H_{11}N_5O_4$	252.0733	N.D.		
Guanidinosuccinic acid	0.17	8.17	47.7	$C_5H_9N_3O_4$	174.0515	N.D.		
4-Pyridone-3-carboxamide-1-β-D-ribonucleoside	0.013	0.57	44.2	$C_{11}H_{14}N_2O_6$	269.0774	N.D.		
Indoxyl sulfate (total)	2.5	108.6	43.2	$C_8H_7NO_4S$	212.0018	212.0019	2.31	2.98
Hippuric acid (free)	5.59	230.7	41.3	$C_9H_9NO_3$	178.0504	178.0504	2.73	4.05
Kynurenic acid	0.029	0.799	27.6	$C_{10}H_7NO_3$	188.0348	N.D.		
Hippuric acid (total)	16.8	398.3	23.8	$C_9H_9NO_3$	178.0504	178.0504	2.73	4.05
p-Cresyl sulfate (free)	0.426	9.31	21.9	$C_7H_8O_4S$	187.0065	N.D.		
Methylguanidine	0.1	1.91	19.1	$C_2H_7N_3$	72.05617	N.D.		
Carboxymethyllysine	1.72	26.5	15.4	$C_8H_{16}N_2O_4$	203.1032	N.D.		
Oxalate	3.33	43.3	13.0	$C_2H_2O_4$	88.98748	N.D.		
N-Methyl-4-pyridone-3-carboxamide	0.26	3.28	12.5	$C_7H_8N_2O_2$	151.0508	N.D.		
Nicotinamide	0.026	0.29	11.2	$C_6H_6N_2O$	121.0402	N.D.		
p-Cresyl sulfate (total)	10.1	111.2	11.0	$C_7H_8O_4S$	187.0065	N.D.		

[a]: Data were obtained from previous reports (Duranton F. *et al. J. Am. Soc. Nephrol.* **23**, 1258-1270, 2012). [b]: Calculated as $[M-H]^-$ ion. [c]: Retention time (min). [d]: peak intensity ratio (KO/WT).
N.D.: No putative corresponding peak was detected.

Table S2. Age of hemodialysis onset and pre-dialysis serum uric acid levels according to ABCG2 status[a].

	ABCG2 function[b]				P value[c]
	100%	75%	50%	≤25%	
N (male/female)	56 (36/20)	63 (48/15)	14 (11/3)	6 (6/0)	
Serum uric acid level [mg/dL][d]					
	7.08±0.96	7.93±0.97	8.26±2.02	9.55[e]	<0.001
Age of hemodialysis initiation [years]					
	59.4±14.8	57.7±14.1	51.4±12.9	49.3±18.9	<0.05

Data are presented as mean±SD.

[a]: Data were obtained from our previous reports (Matsuo H. *et al. Sci Rep.* **6,** 31003;10.1038/srep31003, 2016, Ooyama K. *et al. Progress in Medicine.* **37,** 255-258, 2017 [article in Japanese]) and are shown with slight modifications. [b]: Since ABCG2 has been reported to have two major dysfunctional variants, namely, one half-functional Q141K variant (rs2231142, causative variant of **2* allele) and one non-functional Q126X variant (rs72552713, causative variant of **3* allele), patients were classified into four classes according to the ABCG2 genotype combinations (100% function with the wild-type **1*/**1*; 75% function with **1*/**2*; 50% function with **2*/**2* or **1*/**3*; ≤25% function with **2*/**3* or **3*/**3*) (Matsuo H. *et al. Sci. Rep.* **3,** 2014;10.1038/srep02014, 2013, Matsuo H. *et al. Sci. Rep.* **4,** 3755;10.1038/srep03755, 2014). [c]: Regression analysis. [d]: Patients who were free from uric acid-lowering drugs were analyzed. [e]: SD was not calculated because there were only two patients who were free from uric acid lowering drugs.

Table S3. Gradient program for the comprehensive analysis of anionic compounds.

Time [min]	Solvent A [%]	Solvent B [%]
0	100	0
30	10	90
35	10	90
40	100	0
50	100	0

Table S4. Chromatographic conditions and gradient programs for determination of creatinine, indoxyl sulfate, d4-indoxyl sulfate (d4-IS), and hippuric acid level.

Analytes	Column[a]	Gradient program[b]		
		Time [min]	Solvent A[c] [%]	Solvent B[d] [%]
Creatinine	Amide column[e]	0	2	98
		1.0	2	98
		2.5	30	70
		2.6	60	40
		3.6	60	40
		3.7	2	98
Indoxyl sulfate<br>d4-IS<br>Hippuric acid	C18 column[f]	0	90	10
		2.0	90	10
		7.0	2	98
		8.0	2	98
		8.2	90	10
		10	90	10

[a]: The Column temperature was set at 40°C. [b]: Total flow rate was fixed at 0.3 mL/min. [c]: 0.1% formic acid in water. [d]: 0.1% formic acid in acetonitrile. [e]: ACUITY UPLC® BEH Amide column (1.7 µm, 2.1 × 150 mm, Waters Corp.). [f]: ACUITY UPLC® BEH C18 column (1.7 µm, 2.1 × 100 mm, Waters Corp.).

Table S5. LC-MS/MS monitoring parameters of analytes.

Compound	RT [min]	Ionization mode	Monitor ion [m/z]	
			Precursor	Product
Indoxyl sulfate	3.98	ESI^-	211.98	80.01
d4-IS	3.98	ESI^-	216.01	80.01
Hippuric acid	3.36	ESI^+	180.01	77.03
Penicillin G[a]	5.05	ESI^+	335.12	175.98
Creatinine	3.74	ESI^+	114.03	44.06
Labetalol[b]	3.49	ESI^+	329.11	90.99

[a]: internal standard for indoxyl sulfate, d4-indoxyl sulfate (d4-IS), and hippuric acid. [b]: internal standard for creatinine.
